# Alfalfa mulching improved soil health and ecosystem multifunctionality of *Camellia oleifera* forests on a subtropical karst brae

**DOI:** 10.3389/fmicb.2026.1770805

**Published:** 2026-04-14

**Authors:** Leilei Ding, Birong Gao, Zhongfu Long, Hang Sun, Zhenduan Zhou, Yue Ye, Song Yang, Xia Lei

**Affiliations:** 1 Guizhou Institution of Prataculture, Guizhou Academy of Agricultural Sciences, Guiyang, Guizhou, China; 2 Qiannan Comprehensive Experimental Station of China's Forage Industry Technology System, Guiyang, Guizhou, China; 3 Guizhou Key Laboratory of Agricultural Microbiology, Guizhou Academy of Agricultural Sciences, Guiyang, Guizhou, China; 4 Institute of Subtropical Crops, Guizhou Academy of Agricultural Sciences, Guiyang, Guizhou, China; 5 Guizhou Education University, Guiyang, Guizhou, China; 6 Guizhou Vocational College of Agriculture, Qingzhen, Guizhou, China

**Keywords:** alfalfa mulching, *Camellia oleifera*, ecosystem multifunctionality, microbial assembly, microbial life-history strategy, soil health

## Abstract

**Introduction:**

Despite the economic, ecological, medicinal and horticultural importance as well as national strategic importance of edible oil security, *Camellia oleifera* suffers from low yields due to poor soil health and poor floor managements. Whether alfalfa mulching improves soil health remains largely unknown in *Camellia oleifera* forests.

**Methods:**

This study quantitatively compared soil health and ecosystem multifunctionality under alfalfa mulching and bare control treatments of *Camellia oleifera* forests on a subtropical karst brae.

**Results:**

Alfalfa mulching elevated the relative abundance of beneficial bacteria and fungi, reduced the relative abundance of pathogenic fungi, and enhanced soil health and ecosystem multifunctionality. Understory vegetation diversity, soil microbial function, microbial diversity, microbial life-history strategy, microbial community stability, microbial cross-kingdom network stability, and phylogenetic bin assembly could jointly affect both soil health and ecosystem multifunctionality, however, soil microbial function showed the highest individual effects.

**Discussion:**

This study demonstrates relatively new supports that alfalfa mulching represents a win-win strategy for renovating low-yielding *Camellia oleifera* forests. The strategy works by shifting focus from above-ground vegetation management alone to integrated management of both above- and below-ground microbial communities—specifically through improving soil microbial function via mulching.

## Introduction

*Camellia oleifera* is an evergreen oil crop native to China (Wang Y. et al., 2021; Chen X. et al., 2023; Gao et al., 2024) and recognized by the FAO for its high-quality edible oil (Gao et al., 2024; Qin et al., 2024; Shi X. et al., 2024). Its oil is exceptionally rich in unsaturated fatty acids (nearly 90%) and bioactive compounds that confer antioxidant, anti-inflammatory, and cardiovascular-protective benefits (Liu et al., 2017; Xiao et al., 2017; Liu et al., 2018; Qin et al., 2024). Beyond its nutritional value, by-products of *Camellia oleifera* are utilized in chemicals, fertilizers, and activated carbon (Zhang et al., 2021; Quan et al., 2022; Yang Y. et al., 2025), while the plant itself contributes to ornamental aesthetics (Zhao et al., 2025) and soil and water conservation (Zheng et al., 2008). China currently accounts for over 60% of global cultivation area and more than 95% of total production, with an industrial output exceeding 1,160 billion yuan, supporting about 1.73 million livelihoods (Chen X. et al., 2023; Zheng et al., 2023; Xu et al., 2024; Yang Y. et al., 2025). As cultivation expands globally (Luan et al., 2020; Zhang F. et al., 2022; Gao et al., 2024; Qin et al., 2024; Zhang L. et al., 2024; Zhao et al., 2025), *Camellia oleifera* stands as a cornerstone of sustainable agriculture and holistic health.

Nevertheless, *Camellia oleifera* suffers from low yields (Liu et al., 2018) due to poor soil health (Liu et al., 2017; Wang Y. et al., 2021; Chen et al., 2024; Zhao et al., 2025). Soil health—the capacity of soil to function as a living ecosystem (Lehmann et al., 2020) —and ecosystem multifunctionality—the ability to simultaneously deliver multiple functions (Ding L. et al., 2024) —are critical for sustainable production. However, most research has focused on breeding new varieties and oil functional properties (Xiao et al., 2017; Zhu et al., 2019), with limited attention to improving soil health (Zhang et al., 2025) or multifunctionality (Wang Y. et al., 2021) through management practices. Furthermore, traditional clean tillage, still widely practiced in Chinese orchards (Wang Y.-t. et al., 2024), exacerbates soil degradation by depleting nutrients and reducing enzyme activities (Wang N. et al., 2022; Ma et al., 2025). Conversely, grass mulching has emerged as a promising alternative. Studies show that mulching can curb erosion (Liu et al., 2014), improve soil physicochemical properties (Ding et al., 2018; Wang Z. et al., 2022; Xu et al., 2024; Peng et al., 2025; Zhao et al., 2025) and enzyme activities (Wang et al., 2020b; Wang N. et al., 2022; Wang X. et al., 2022; Wang Y. et al., 2022; Yang et al., 2024), elevate plant survival rate (Lin and Wang, 2024), enhance biodiversity (Gómez et al., 2017) and fruit yield (Wang Q. et al., 2021; Peng et al., 2025; Zhao et al., 2025). Grass mulching can also shift soil microbial community assembly (Wang et al., 2020b; Ma et al., 2021), diversity (Wang et al., 2020b; Castellano-Hinojosa et al., 2023; Bajiu et al., 2024; Wang S. et al., 2024; Xu et al., 2024), life-history strategy (Yang et al., 2020; Chen et al., 2021; Yang T. et al., 2023), community stability, and network stability (Ding et al., 2026), as well as improve soil microbial function (Li et al., 2022). Alfalfa, a nitrogen-fixing legume, may be effective due to its impact on soil microbiota. However, the mechanistic pathways through which alfalfa mulching influences soil health and ecosystem multifunctionality remain unclear in *Camellia oleifera* forests.

Specifically, while mounting evidence suggests that soil health (Saleem et al., 2019; Tahat et al., 2020; Pereira et al., 2021; Luo et al., 2022; Trinchera et al., 2022; Cheng et al., 2023) and multifunctionality (Xiao and Veste, 2017; Bashir et al., 2022; Zhang K. et al., 2022; Chen et al., 2023a; Gao et al., 2023; Li J. et al., 2023; Xu P. et al., 2023; Ding L. et al., 2024; Gong et al., 2024; Wang W. et al., 2024; Yang C. et al., 2025) are shaped by multiple plant and microbiota factors—no study has systematically compared the relative contributions of understory vegetation diversity, microbial diversity, function, life-history strategies, community stability, cross-kingdom network stability, and phylogenetic bin assembly processes in *Camellia oleifera* ecosystems. Critically, it is unknown whether alfalfa mulching enhances soil health and multifunctionality primarily through: (i) understory vegetation diversity, (ii) direct improvements in soil physicochemical properties, (iii) alterations in microbial diversity and community stability, (iv) shifts in microbial life-history strategies (e.g., copiotroph vs. oligotroph), (v) changes in cross-kingdom microbial network stability and phylogenetic bin assembly processes, or (vi) improvements in microbial functions.

Therefore, this study aimed to: (1) assess the effects of alfalfa mulching on soil health and ecosystem multifunctionality in *Camellia oleifera* forests on a subtropical karst brae; (2) quantify the relative contributions of understory vegetation diversity, microbial diversity, microbial life-history strategy, microbial community stability, cross-kingdom network stability, and phylogenetic assembly processes to soil health and multifunctionality; and (3) identify the potentially primary mechanistic pathways through which alfalfa mulching exerts its effects. We hypothesized that: (H1) alfalfa mulching would significantly enhance both soil health and ecosystem multifunctionality compared to bare control since we were informed by the improvement of soil physiochemical and enzymic properties (Ahmad et al., *2018*; Bajiu et al., 2024; Elhaddad et al., 2024) and yield and biomass (Ozturkmen et al., 2020); and (H2) alfalfa mulching would improve soil health and multifunctionality primarily by shifting microbial functions toward more beneficial and less harmful ones. By systematically comparing the above biotic drivers, this study provides relatively new insights into the mechanisms underlying soil health and ecosystem multifunctionality improvement and offers guidance for orchard floor management strategies aimed at collaboratively enhancing both soil health and ecosystem multifunctionality in *Camellia oleifera* plantations.

## Materials and methods

### Research region description and experimental design

The research area (N25°13′58″, E106°09′6″, elevation 850 m, slop 20°) was located in Nonglin Village, Wangmo County, Qianxinan Buyi and Miao Autonomous Prefecture, Guizhou plateau of China. The region is characterized by a typical subtropical monsoon humid climate, with a mean annual temperature of 19 °C mean annual precipitation of 1,237 mm, average annual sunshine duration of 1,402 h, and average frost-free period of 340 days (Ding et al., 2026). The soils of the region are shallow, rocky karst soils, which are highly susceptible to erosion under bare conditions. The research was conducted in this county because the county has developed extensive *Camellia oleifera* cultivation (covering an area of ~150,000 acres) as a key economic industry and represents renovation needs of low-yield forests. This study, however, was not sampled across the entire region. Instead, a single, uniform slope (20°) was selected to strictly control for topographic and edaphic variables to minimize the differences stemmed from non-design source (e.g., climate and soil background). Sixteen plots were established to represent eight alfalfa mulching plots (AM) and eight bare control plots (BC) in the *Camellia oleifera* forests on the uniform slope (20°). Weeds were manually cut for both the AM and BC plots before sowing (1.5 g alfalfa seed per m^2^) and during the seedling stage of alfalfa.

### Experimental sampling

First, soil temperature was measured using soil thermometers (Shenzhen Lixinda Electronic Technology Co., Ltd., China) at 5 cm soil depth and ground surface temperature and humidity were measured using electronic temperature and humidity meters for each plot (0.5 × 0.5 m^2^) during early June 2025. Second, the number of understory vegetation were counted, five heights of understory vegetation were randomly measured using rulers (precision of 0.1 cm) to obtain the average height of understory vegetation, and the aboveground biomass of understory vegetation were collected to the ground level using stainless-steel scissors. Third, the gases of CO_2_, CH_4_, and N_2_O emission from soils were collected using static chambers (inner diameter: 23 cm, height: 30 cm). Each chamber was equipped with a built-in fan (1,500 rpm, with seven 9-cm blades) to ensure thorough air mixing. Gas samples were extracted from the center of the chamber using separate 50-ml syringes immediately upon chamber closure and again after 1 h. All extracted gas samples were subsequently transferred and stored in gas bags (Ningbo Hongpu Experimental Technology Co., Ltd., China). Finally, a total of 37 topsoil cores (0–5 cm depth) were evenly collected across the plot using separate stainless-steel ring cutters (inner diameter: 5 cm; height: 5 cm). Vegetation roots were carefully extracted using 10 soil cores, washed over a 2-mm sieve. Both aboveground understory biomass and roots were dried at 105 °C to kill, followed by 65 °C drying to constant weight in a precision drying oven (BPG-9140A, Shanghai Yiheng Scientific Instrument Co., Ltd., China). The resulting biomasses were recorded as understory vegetation aboveground biomass and vegetation root biomass, respectively. One intact soil core was used to determine bulk density, capillary porosity, and non-capillary porosity. The remaining 26 soil cores were thoroughly homogenized and passed through a separate 2-mm sieve. The sieved soil was then divided into three subsamples for the determination of soil physical and chemical properties, enzyme activities, and DNA extraction for soil bacterial and fungal community analysis. Throughout the procedure, separate sterile medical gloves (Yiwu Yintongmei Medical Technology Co., Ltd., China) were used to prevent cross-contamination. A detailed description on the collection process of soil related samples have also been provided in our recent study (Ding et al., 2026).

### Determination of soil physicochemical properties and vegetation carbon

Soil bulk density, porosity, and water content were measured using the oven-drying method (Ding et al., 2026). Soil pH was analyzed potentiometrically. Total nitrogen content was quantified by Kjeldahl digestion. Ammonium and nitrate nitrogen were extracted with potassium chloride. Total phosphorus was determined by the molybdenum antimony anti-colorimetric method, while available phosphorus was assessed using double acid extraction. Total and available potassium were analyzed by flame photometry. Total calcium and magnesium were measured after digestion with HCl–HNO₃–HClO₄. Dissolved organic carbon was extracted with water. Microbial biomass carbon was determined by the fumigation method and using potassium dichromate and concentrated sulfuric acid with external heating. Microbial biomass nitrogen and phosphorus were analyzed via chloroform fumigation followed by potassium sulfate extraction and UV spectrophotometry, respectively. These parameters were used to estimate microbial carbon use efficiency with the R function “MicrobUIQ”^1^. Soil organic carbon content was analyzed with a TOC analyzer, while inorganic carbon was measured by volumetric titration. The storages of soil organic and inorganic carbon were calculated by multiplying their respective concentrations by the soil bulk density and the height of the ring cutter. Concentrations of CO₂, CH₄, and N₂O were determined via gas chromatography. The global warming potential (kg CO₂-equivalents per hectare on a 100-year scale) was computed as the sum of CO₂, 27.9 times CH₄, and 273 times N₂O (Ansari et al., 2023). Carbon content in understory vegetation aboveground biomass, root biomass, and soil organic carbon were analysed using the potassium dichromate concentrated sulfuric acid external heating method (Lu et al., 2025). Corresponding carbon storages were derived by multiplying carbon content by the respective biomass.

### Determination of soil enzyme activities

Enzyme activities including polyphenol oxidase, peroxidase, and sucrase were measured spectrophotometrically. *β*-1,4-Glucosidase, cellobiohydrolase, β-1,4-N-acetylglucosaminidase, β-1,4-xylosidase, *α*-1,4-glucosidase, leucine aminopeptidase, and acid phosphatase were assayed using fluorometric methods. Microbial carbon and phosphorus limitations were evaluated via enzyme vector analysis (Ding et al., 2023). Soil organic carbon decomposition and lignocellulose index was calculated using a microbial enzyme allocation framework (Hill et al., 2014).

### Soil DNA extraction, amplicon sequencing, and bioinformatics processing

Soil DNA was extracted using commercial isolation kits. The bacterial 16S V3–V4 region and fungal ITS1 region were amplified and sequenced on an Illumina NovaSeq platform (Lu et al., 2025). Raw sequences were processed using the DADA2 pipeline to generate amplicon sequence variant (ASV) tables, after chimera removal with UCHIME. Taxonomic assignment was performed in QIIME2 against the SILVA (bacteria) and UNITE (fungi) databases (Lu et al., 2025). Phylogenetic trees were constructed using QIIME2. Fungal functional guilds were assigned with the “FUNGuildR” package in R (Ding et al., 2023); only “probable” and “highly probable” assignments were retained for downstream analysis.

### Metagenomic sequencing and bioinformatic analysis

Metagenomic DNA was fragmented to ~350 bp using a Covaris ultrasonicator, followed by library preparation and sequencing on an Illumina platform (PE150 mode). Raw reads were quality-filtered with Fastp. Metagenome assembly was performed with MEGAHIT, and open reading frames (ORFs) on assembled scaftigs were predicted using MetaGeneMark. A non-redundant gene catalogue was constructed with CD-HIT at 95% identity threshold.

For taxonomic annotation, unigenes were aligned against the Micro_NR database using DIAMOND, and species-level assignments were determined via MEGAN. To address our specific research focus on orchard soil health, plant beneficial and harmful bacteria were further identified at genus and species levels based on the database of Li P. et al. (2023).

### Dissection of soil microbial phylogenetic bin assembly mechanism

Soil microbial phylogenetic bin-based assembly mechanisms were quantified using the iCAMP (phylogenetic bin-based null model analysis) package (Ning et al., 2020) based on the amplicons data, since microbial assembly were suggested to be more sensitive to management (Neal et al., 2021). Thirty environmental factors including plant and soil properties were used (Table. S1). Before analyzing, we optimized two key parameters (“ds”: phylogenetic signal threshold; “bin.size.limit”: minimal bin size) that affect the analysis results by testing at different levels of “ds” and “bin.size.limit.” By doing so, the optimized “ds” and “bin.size.limit” results in the highest number of bins with significant phylogenetic signal and relatively high average correlation coefficient (meanR) within bins (Ning et al., 2020).

### Construction of cross-kingdom microbial network

Network analysis offers valuable insights into potential microbial interactions (Matchado et al., 2021) and network resistance (Mu et al., 2021; Gao et al., 2023). To enhance network reliability, ASVs with a total relative abundance below 0.00001 or detected in fewer than three-eighths of the samples were excluded from the networks. The networks were constructed using the “igraph” package, retaining only edges with a Pearson correlation coefficient absolute value > 0.8 and a Benjamini-Hochberg corrected *p*-value < 0.001 (Wang P. et al., 2022). Positive and negative cohesion, as well as network stability, were calculated based on the amplicon data following the methods described by Herren and McMahon (2017) and Yuan et al. (2021).

### Soil health quantification

Principal component analysis is used in developing a mini dataset (Table. S2) of key soil health indicators (Li et al., 2024; Lu et al., 2024b; Zhang J. et al., 2024) by performing the function “principal” in R.4.0.5, with the chosen principal component eigenvalues>1 (Lu et al., 2024a) and excluded indicator that not in one tenth of the highest load (Lu et al., 2024b). The included soil health indicators chiefly mirror soil health (Bagnall et al., 2023) and are frequently applied in soil health quantifications (Pagliai and Vignozzi, 2006; Zhang M. et al., 2022).

Two kinds of scoring function are employed: “more is better” (Understory vegetation aboveground biomass, soil noncapillary porosity, soil water content, soil total potassium, soil total magnesium, microbial biomass phosphorus, NAG, αG, bacterial Chao1, soil organic carbon, soil total nitrogen, soil total calcium, soil slowly potassium, fungal Shannon, fungal Simpson, ectomycorrhizal, and fungal symbiotroph) (Wang et al., 2020b; Zhang et al., 2023; Lu et al., 2024a; Lu et al., 2024b); “less is better” (soil temperature, soil bulk density, plant pathogen, CH_4_ flux, and N_2_O flux) (Zeraatpisheh et al., 2020; Lu et al., 2024a). The frequently used scoring approach are used to score the included soil health indicators.

$L_{\text{more}}=\{\begin{array}{c} 0,x\leq L\\ \frac{x-L}{H-L},L<x<H\\ 1,x\geq H \end{array}\}$ and $L_{\text{less}}=\{\begin{array}{c} 1,x\leq L\\ \frac{H-x}{H-L},L<x<H\\ 0,x\geq H \end{array}\}$ (Li et al., 2024).

Four most frequently used integration approach, including equal weighted addition (Zhang et al., 2023), PCA weighted addition (Lu et al., 2024b), nemoro soil health (Zahedifar, 2023), and SQI-area approach (Kuzyakov et al., 2020), are used to integrate the scores into a composite soil health index. Based on the above approaches, this study results in total four soil health indexes.

### Ecosystem multifunctionality quantification

Sixty functional variables are included in multifunctionality quantification, including, temperature and wetness regulation functions (soil temperature, ground surface temperature, ground surface humidity, CO_2_ flux, CH_4_ flux, N_2_O flux, global warming potential, and modeled respiration) (Ding L. et al., 2024; Mamabolo et al., 2024), nutrient supporting functions (Garland et al., 2020; Zhang et al., 2021; Ding L. et al., 2024; Mamabolo et al., 2024) (understory vegetation aboveground biomass carbon vegetation root biomass carbon, understory vegetation aboveground biomass carbon storage, vegetation root biomass carbon storage, soil organic carbon, soil inorganic carbon, soil organic carbon storage, soil inorganic carbon storage, soil alkali-hydrolyzable nitrogen, soil ammonium nitrogen, soil nitrate nitrogen, soil available phosphorus, soil acid soluble potassium, soil available potassium, soil slowly potassium, microbial biomass carbon, microbial biomass nitrogen, microbial biomass phosphorus, soil dissolved organic carbon, βGC, CBH, NAG, βX, αG, LAP, ACP, POX, PER, Urease, Sucrase, microbial carbon use efficiency, and organic carbon decomposition, plant beneficial bacteria, fungal symbiotroph, arbuscular mycorrhizal, and ectomycorrhizal), productivity (Ding L. et al., 2024; Feng et al., 2024) (understory vegetation aboveground biomass, and vegetation root biomass), biodiversity reservoir function (bacterial Chao1, bacterial Shannon, bacterial Simpson, fungal Chao1, fungal Shannon, fungal Simpson, bacterial community stability, fungal community stability, and understory vegetation species richness) and harmful microorganisms control (phytopathogen bacteria, fungal pathotroph, plant pathogen fungi, fungal parasite, and plant parasite fungi). Soil temperature, ground surface temperature, CO_2_ flux, CH_4_ flux, N_2_O flux, global warming potential, organic carbon decomposition, modeled respiration, phytopathogen bacteria, fungal pathotroph, plant pathogen fungi, fungal parasite, and plant parasite fungi are transformed via applying t(x) = max(x) − x such that high values signify good states. The functions are standardized using R function “standardizeUnitScale” (Ding L. et al., 2024). The multifunctionality is quantified using three wide used approaches, that is, averaging, entropy (using R function “getMF”), and threshold approaches (using R function “getFuncsMaxed” in R v3.6.1), which cover various facets of multifunctionality (Ding L. et al., 2024).

### Statistical analysis

The Shapiro and Levene tests are applied to determine the normal distribution and homoscedasticity. If the requirements of normality and homogeneity were met, the R function “aov” and “t.test” were used, or “kruskal.test” and “wilcox.test” were used in R v3.6.1. Spearman correlation analysis was applied to explore the relationships of soil health, ecosystem multifunctionality with microbiota and plant, and Pearson correlation analysis was applied to explore the relationships of soil health with ecosystem multifunctionality through performing the “psych” package in R3.6.3and “GGally” in R4.3.0, respectively. Redundancy analysis-based hierarchical partitioning with 10,000 permutations was used to separate the individual effects of different facets of biotic driving forces on soil health and ecosystem multifunctionality of *Camellia oleifera* forests using the R “rdacca.hp” package (Lai et al., 2022).

## Results

### Alfalfa mulching elevated soil health and ecosystem multifunctionality

**Figure 1:**
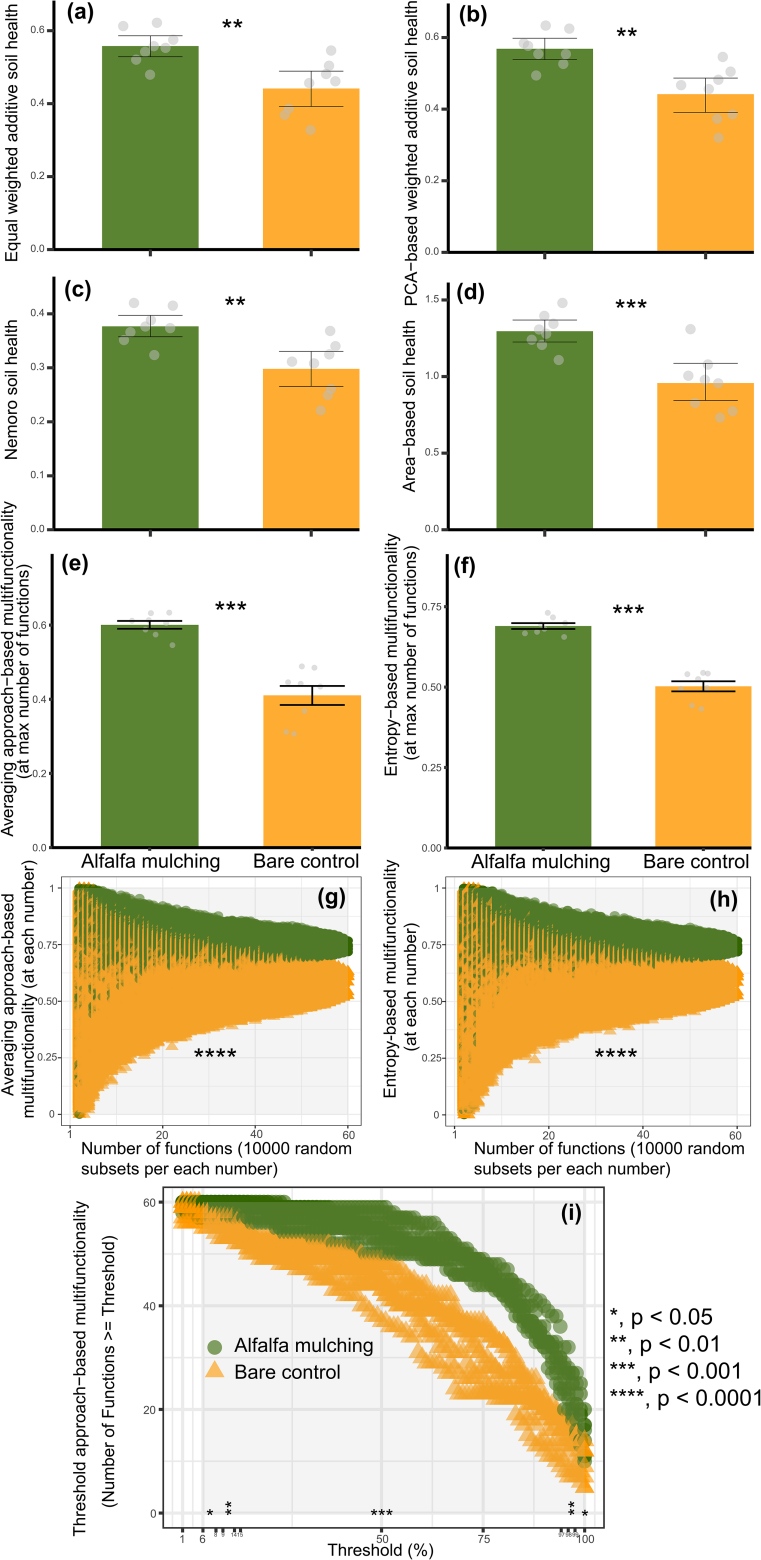
Soil health and ecosystem multifunctionality under alfalfa mulching (AM) and bare control (BC). **(a)** Equal weighted additive soil health; **(b)** principal component analysis (PCA)-based weighted additive soil health; **(c)** nemoro soil health; **(d)** area-based soil health; **(e,f)** the averaging-based multifunctionality; **(g,h)** entropy-based multifunctionality; **(i)** multiple threshold approach-based multifunctionality.

To enhance the robustness of our findings, we assessed soil health and ecosystem multifunctionality using multiple complementary methods. Across all methods—including equal-weighted additive, PCA-weighted additive, Nemoro, and area-based indices—alfalfa mulching showed significantly higher soil health relative to the bare control (ANOVA and *t*-test, *p* < 0.01; Figures 1a–d). Similarly, ecosystem multifunctionality was consistently higher under alfalfa mulching relative to under the bare control, as demonstrated by averaging-based, entropy-based, and multiple threshold-based methods (Kruskal–Wallis and Wilcoxon tests, *p* < 0.05; Figures 1e–i).

### Alfalfa mulching shifts soil microbial phylogenetic bin assembly

To investigate microbial community assembly mechanisms, we applied the recently published iCAMP method (Zheng et al., 2022) to phylogenetic bins (Ning et al., 2020) derived from 13,532 bacterial and 9,449 fungal ASVs. Optimal phylogenetic signal thresholds and minimum bin sizes were set at 0.2 and 59 for bacteria (Supplementary Figure S1), and 0.2 and 32 for fungi (Supplementary Figure S2), yielding 104 bacterial and 147 fungal bins.

**Figure 2:**
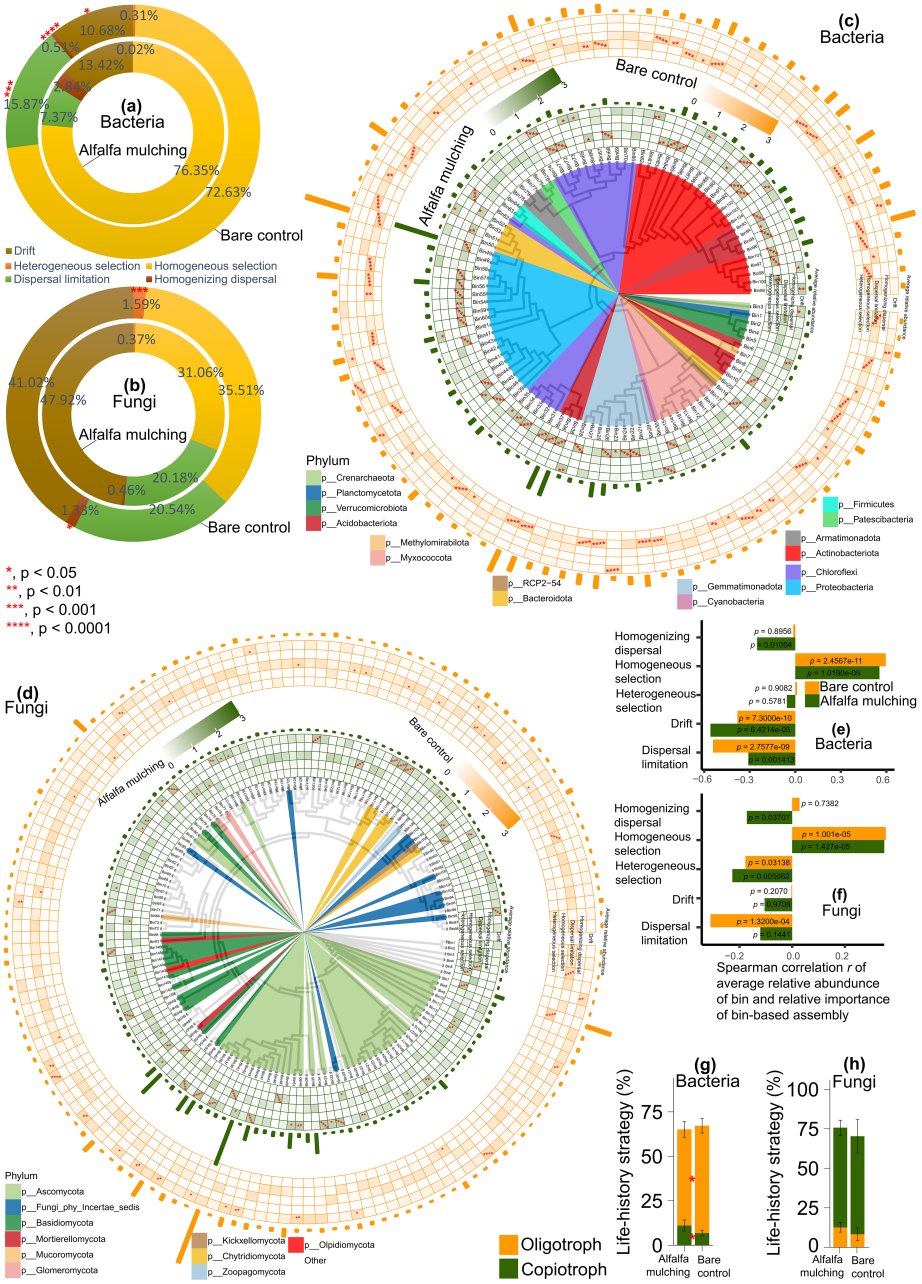
Phylogenetic bin-based assembly mechanisms in bacteria and fungi under alfalfa mulching and bare control. **(a,b)** Bacterial **(a)** and fungal **(b)** community assembly under alfalfa mulching and bare control. **(c,d)** The relative relevance and relative importance of several assembly processes in each bin of bacterial **(c)** and fungal **(d)** community under alfalfa mulching and bare control. **(e,f)** Spearman correlation analysis results of average relative abundance of bin and relative importance of bin-based assembly. **(g,h)** Copiotroph-oligotroph life-history strategy of bacterial **(g)** and fungal **(h)** community under alfalfa mulching and bare control.

Homogeneous selection accounted for 76.4 and 72.6% of community assembly processes under alfalfa mulching and bare control, respectively (Figure 2a). Alfalfa mulching showed the significantly lower relative importance of dispersal limitation (Cohen’s *d* = −5.06, bootstrapping *p* < 0.001), while the higher relative importance of homogenizing dispersal (Cohen’s *d* = 7.25, *p* < 0.001) and drift (Cohen’s *d* = 2.91, *p* < 0.05). At the bin level, homogeneous selection showed the highest relative importance in 42 and 39 bins under mulching and control, respectively, whereas dispersal limitation showed the highest relative importance in only 2 bins under mulching versus 17 bins under control (Figure 2c). Homogenizing dispersal showed the highest relative importance in a single bin under mulching and none under control, while drift showed the highest relative importance in 17 and 6 bins, respectively.

In contrast, drift accounted for 47.9 and 41.0% of community assembly processes under mulching and control (Figure 2b). Alfalfa mulching showed the significantly lower relative importance of heterogeneous selection (Cohen’s *d* = −3.03, *p* < 0.001) and homogenizing dispersal (Cohen’s *d* = −1.70, *p* < 0.05). Bin-level analysis revealed that drift showed the highest relative importance in 33 bins under mulching and 18 under control, while dispersal limitation showed the highest relative importance in 16 and 15 bins, and homogeneous selection showed the highest relative importance in 7 and 4 bins, respectively (Figure 2d).

Across both communities, average relative abundance of bins was positively correlated with the relative importance of homogeneous selection (Spearman’s r = 0.35–0.60, *p* < 0.001), but negatively correlated with dispersal limitation and drift (Figures 2e,f).

### Alfalfa mulching shifts soil microbial life-history strategy and functions

Alfalfa mulching exhibited significantly higher bacterial copiotroph strategies (Wilcoxon test, *p* < 0.05) and lower bacterial oligotroph strategies (*t*-test, *p* < 0.05) relative to the bare control (Figure 2g). In contrast, no significant shifts were observed in fungal copiotroph or oligotroph strategies (*t*-test, *p* > 0.05; Figure 2h).

**Figure 3:**
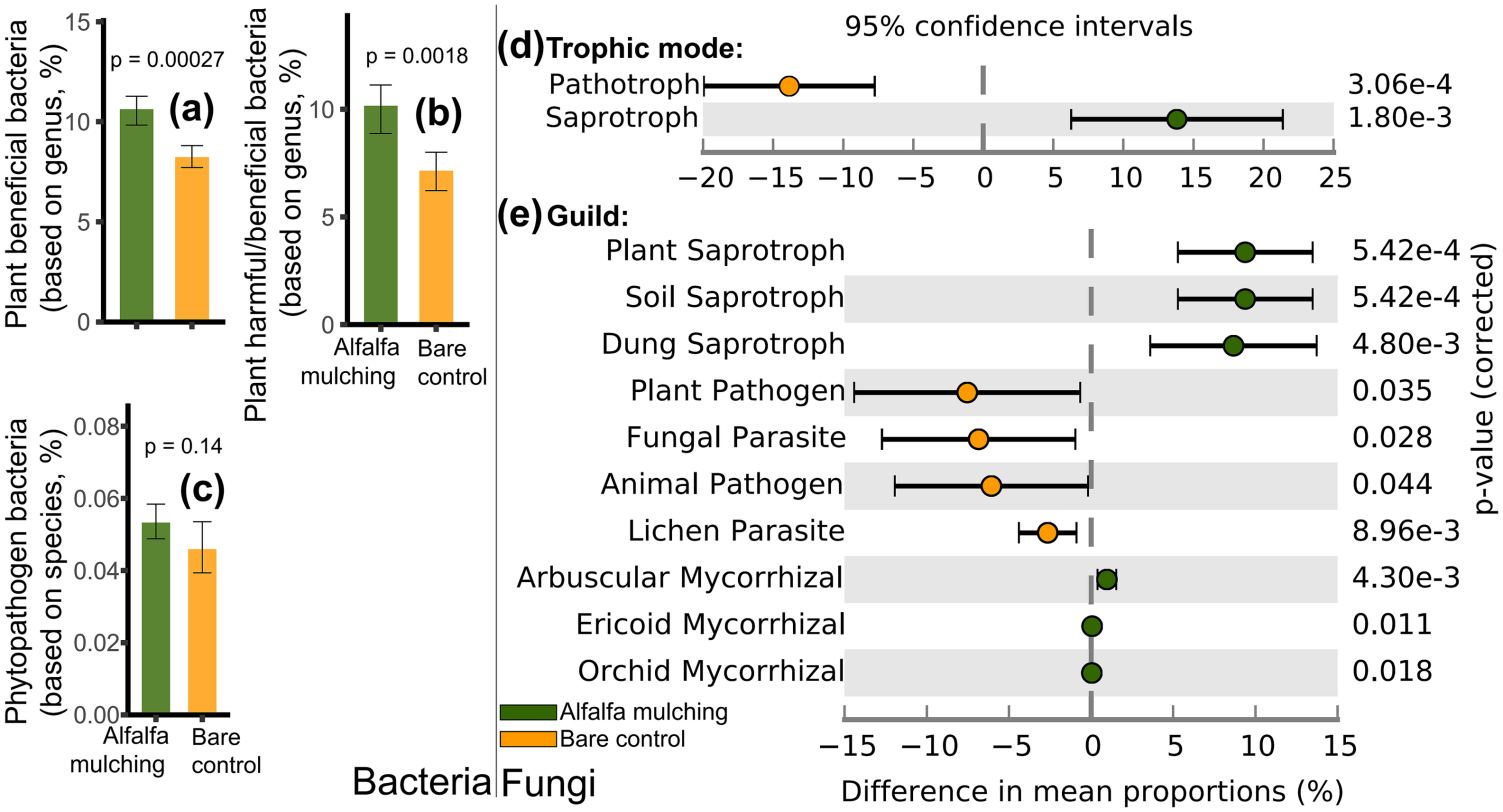
Beneficial and harmful bacteria **(a-c)** and fungal **(d-e)** functions under alfalfa mulching and bare control. *p < 0.05; **p < 0.01.

Functional assignment further revealed that alfalfa mulching enriched multiple beneficial microbial groups, including plant-beneficial bacteria (*t*-test, *p* < 0.001; Figure 3a), arbuscular, ericoid, and orchid mycorrhizal fungi (*p* < 0.05), and diverse saprotrophic fungi (plant, soil, dung; *p* < 0.01; Figure 3b). Conversely, mulching suppressed several pathogenic and parasitic groups, including fungal pathotrophs, plant pathogens, fungal and animal parasites, and lichen parasites (*p* < 0.05). No significant difference was detected for phytopathogenic bacteria (*p* > 0.05; Figure 3a).

### Alfalfa mulching changes soil microbial cross-kingdom network

**Figure 4:**
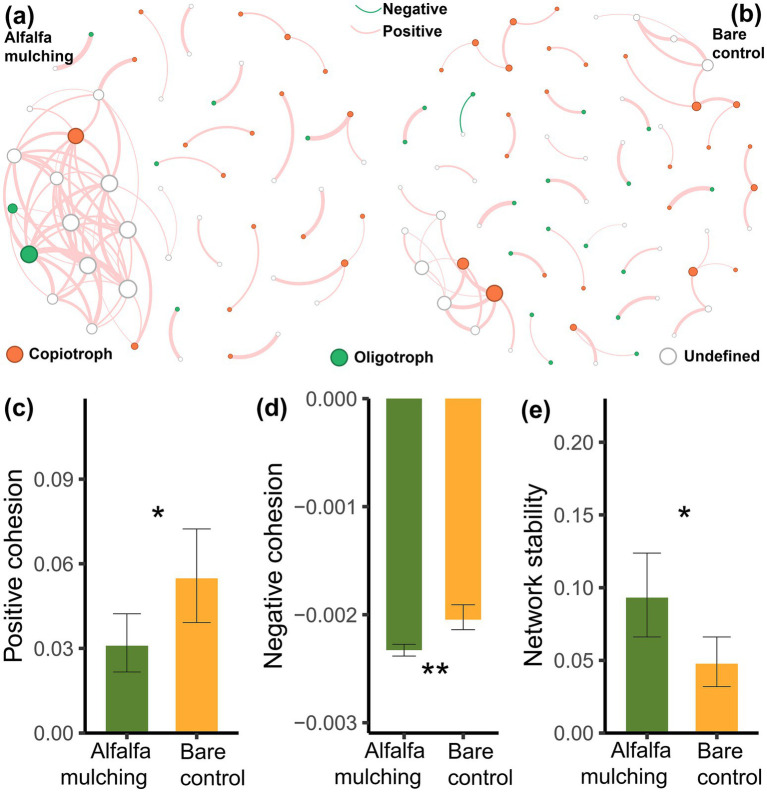
Cross-kingdom (bacteria-fungus) networks **(a,b)**, network cohesion **(c,d)**, and stability **(e)** under alfalfa mulching and bare control. **(a)** Cross-kingdom network under alfalfa mulching; **(b)** Cross-kingdom network under bare control. A node represents an ASV. Links signify the significant Pearson correlation absolute values > 0.8 and BH-corrected *p* < 0.001. Red links signify synergism between ASVs, whereas blue links signify antagonism between ASVs.

Alfalfa mulching exhibited the structure distinct network of soil microbial cross-kingdom compared with the bare control (Figures 4a,b). Compared with the bare control, alfalfa mulching exhibited significantly lower network positive and negative cohesion (*t*-test, *p* < 0.05, Figures 4c,d), while higher network stability (*t*-test, *p* < 0.05, Figure 4e).

### Relationships of soil health and ecosystem multifunctionality with microbiota, soil and plant

Soil health and ecosystem multifunctionality were significantly associated with vegetation, soil, and microbial drivers (*p* < 0.05). These relationships were basically consistent across all calculation methods.

## Discussion

### Linking soil health to microbiota and plant

Many studies concluded that the low yielding and poor quality orchards, including most of *Camellia oleifera* forests, suffered from low soil fertility (Li et al., 2019; Dung et al., 2022; Chen et al., 2023b) such as low nutrition (Liu et al., 2017; Wang Q. et al., 2021) and drought (Wang Q. et al., 2021). In this study, alfalfa mulching increased soil microbial biomass carbon (Ding W. et al., 2024; Ding et al., 2026), nitrogen, and phosphorus (Supplementary Figure S3), capillary porosity, and water content (Chen et al., 2019; Ma et al., 2025) as well as decreased soil bulk density (Shi X. et al., 2024; Peng et al., 2025) and temperature (Ding et al., 2026). These were in line with previous findings from other studies (Xu H. et al., 2023; Lv et al., 2025; Peng et al., 2025; Zhao et al., 2025). Furthermore, phosphorus is a major constraint nutrient for plant growth and yield (Liu et al., 2017; Liu C. et al., 2021) in Southern China (Lv et al., 2025; Zhou et al., 2025), especially in *Camellia oleifera* forests (Wu F. et al., 2021; Chen et al., 2024). Alfalfa mulching boosted the exogenous input of soil organic matter and enzyme activities (Wang N. et al., 2022; Ma et al., 2025). The increased soil dissolved organic carbon, phenol oxidase, *β*-glucosidase (Shi C. et al., 2024), cellobiohydrolase (Ding et al., 2026), β-1,4-N-acetylglucosamine (Supplementary Figure S3), urease (Zhao et al., 2025) (Supplementary Figure S3), and acid phosphatase activities (Wang N. et al., 2022; Gu et al., 2025) (Supplementary Figure S3) alleviated microbial carbon and phosphorus limitation (Ding et al., 2026). These furthermore promoted organic matter degradation (Ding et al., 2026) and soil nutrient mineralization (Wang N. et al., 2022), and consequently increased available nitrogen and phosphorus (Shi C. et al., 2024; Liu et al., 2025; Lv et al., 2025; Zhou et al., 2025; Ding et al., 2026). These improvements could increase root growth, fruit growth (Shi X. et al., 2024), fruit (Peng et al., 2025), oil yields and unsaturated fatty acid content (Chen et al., 2023b), thus have important implications for improvements in *Camellia oleifera* growth, oil yield and quality. Therefore, from the perspective of soil physicochemical health, this study recommends the use of alfalfa mulching.

**Figure 5:**
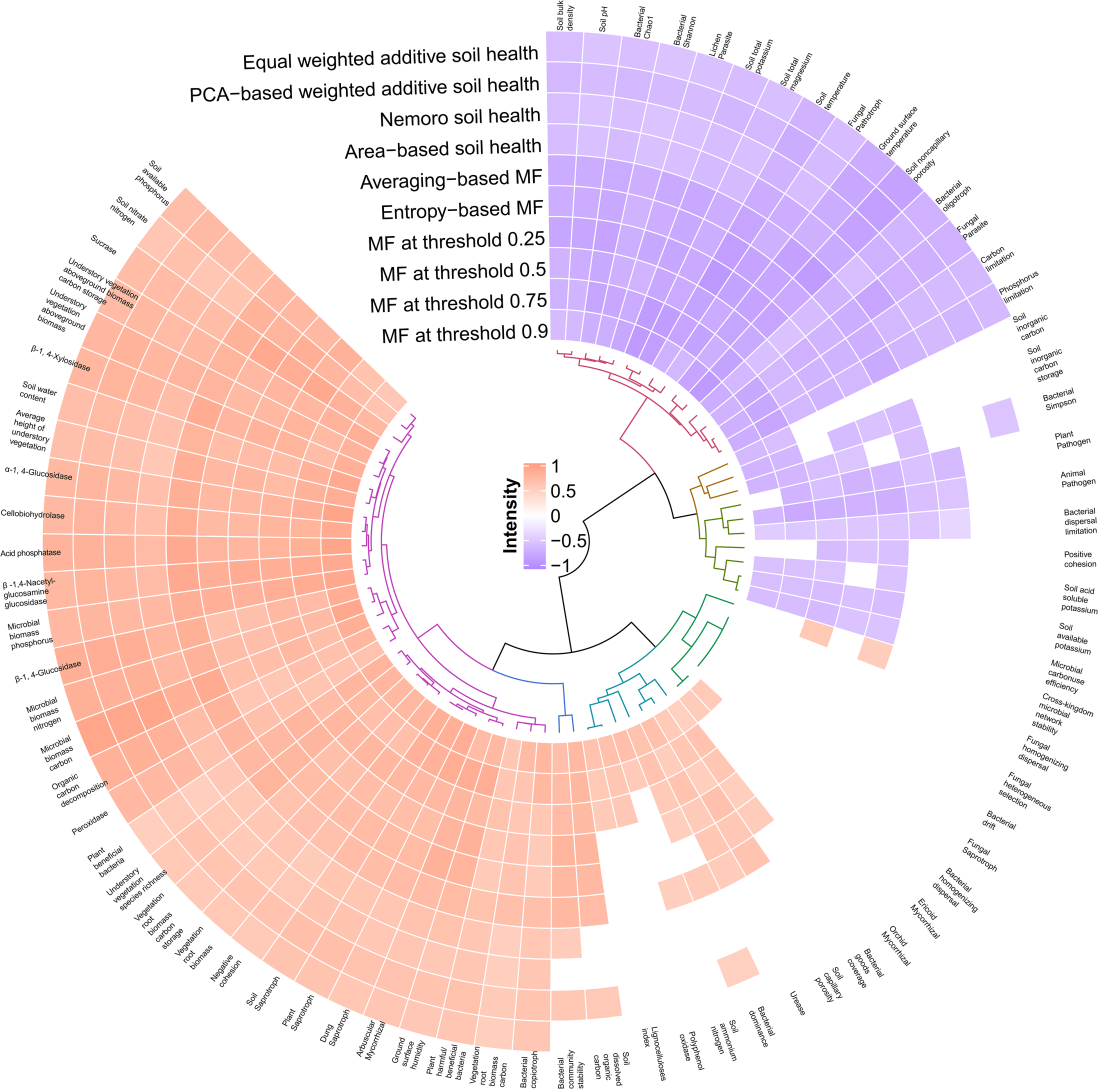
Spearman correlation relationships of soil health and ecosystem multifunctionality with microbiota, soil, and plant. Blank cells denote correlations lacking statistical significance (*p* > 0.05).

Furthermore, this study demonstrated that alfalfa mulching improved soil microbial health. Alfalfa mulching elevated fungal saprotroph, plant saprotroph, soil saprotroph, and dung saprotroph fungi (Figure 3). The increased saprophytic fungi was a result of increased litter residues (Yang et al., 2024). Saprophytic fungi secrete various enzymes (Spearman *r* = 0.59–0.86, *p* < 0.01) into soils to decompose plant residues (Yang et al., 2024) and replenish soil organic matter, which is crucial to accelerate the decomposition of organic matter and release of available nutrients (Hu L. et al., 2022). Moreover, previous studies suggested that grass mulching alters the fungal composition, increased activity of arbuscular mycorrhizal fungi (Turrini et al., 2017), propagules (Wang et al., 2016), spore density (Jiao et al., 2019), colonization rate of roots (Jiao et al., 2019; He et al., 2023), promoting growth of trees (Ding et al., 2021). The alfalfa mulching-induced elevated arbuscular mycorrhizal fungi (Figure 3b) could enhance soil mycorrhizal inoculum potential (Turrini et al., 2017). *Camellia oleifera* is a mycorrhizal plant with up to 42% of the roots colonized by arbuscular mycorrhizal fungi (Liu R.-C et al., 2021). Arbuscular mycorrhizal fungi are very important beneficial microbial symbionts for most (80%) terrestrial plants (Wang et al., 2016; Turrini et al., 2017). They not only improves host plant uptake and translocate of soil nutrients, especially phosphate (Wang et al., 2016; Wu F. et al., 2021) and nitrogen (Wang Z. et al., 2022) through large external mycelia (Turrini et al., 2017), resulting in enhanced plant nutrition and health (Turrini et al., 2017), but also improves biotic (e.g., disease) and abiotic stresses (e.g., drought) resistance of host plants (Wang et al., 2016; Wang Z. et al., 2022). Both contributes to soil quality (Wang et al., 2016) and the production of safe and high-quality food (Turrini et al., 2017). In addition to multiple potential benefits of arbuscular mycorrhizal fungi to plant and soil health (Rodriguez-Paiatsyka et al., 2025) (Spearman *r* = 0.59–0.64, *p* < 0.05, Figure 5), this study demonstrated that alfalfa mulching not only facilitated plant beneficial bacteria (Qian et al., 2018; Yang et al., 2024) (Figure 3a), but also inhibited fungal pathotroph (Ma et al., 2021), plant pathogen fungi, fungal parasite, animal pathogen fungi, and lichen parasite fungi (Figure 3b). Pathogenic fungi were the primary causes of diseases in *Camellia oleifera* with the up to 80% drop of flowers and fruits of *Camellia oleifera* (Chen X. et al., 2023), severe economic losses and posing a huge threat to *Camellia oleifera* industry (Wang et al., 2020a). More beneficial (Wu B. et al., 2021; Yang et al., 2024) and fewer pathogenic microorganisms (Li et al., 2019; Wang Z. et al., 2022) suggested that alfalfa mulching shifted soil microbial composition to more healthy one, likely benefiting to soil (Spearman *r* = 0.51–0.57, *p* < 0.05 for plant beneficial bacteria; Spearman *r* = −0.77––0.41, *p* < 0.05 for pathogenic fungi, Figure 5) and *Camellia oleifera* health.

**Figure 6:**
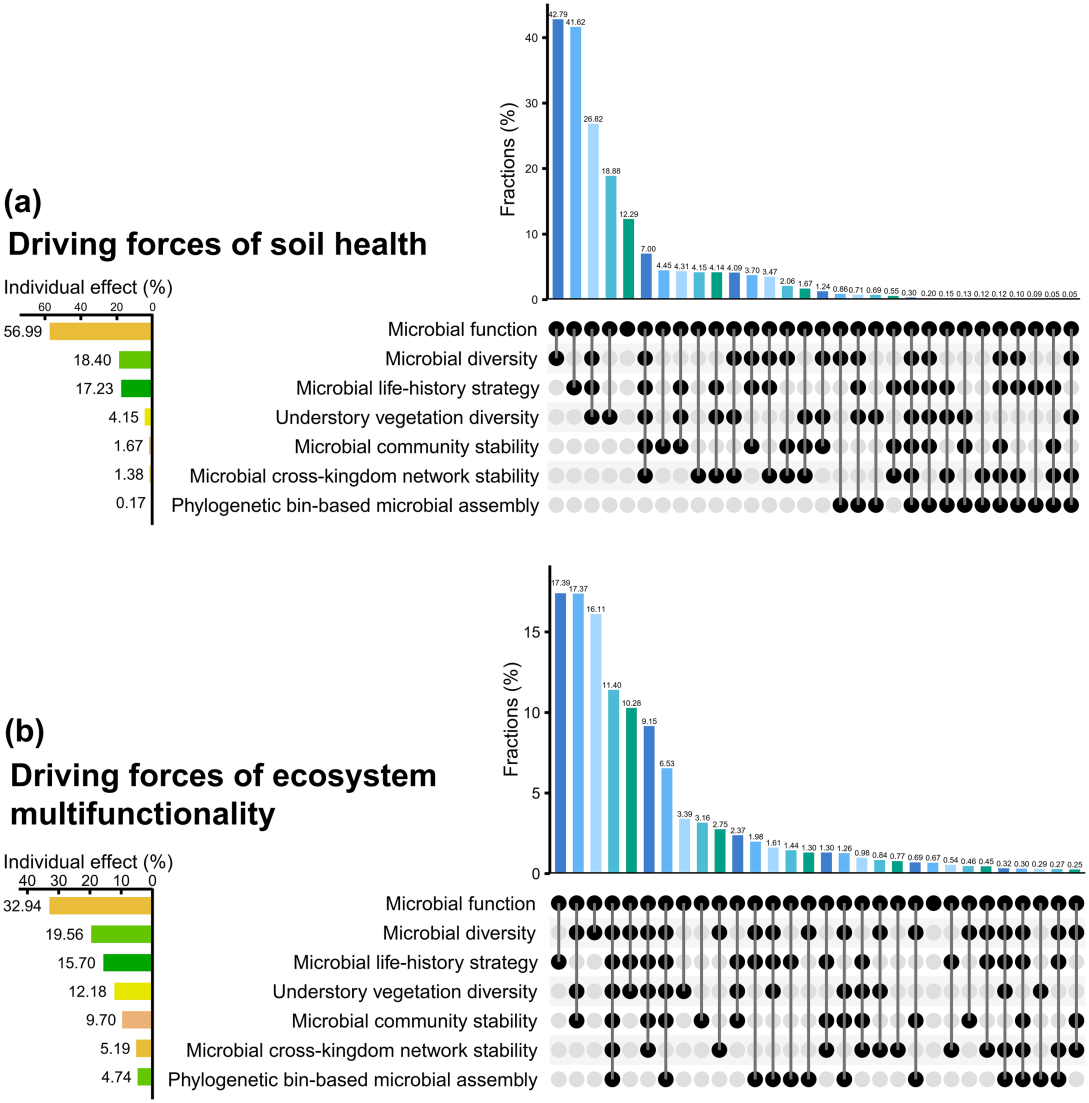
The relative effects of different facets of biotic driving forces on **(a)** soil health and **(b)** ecosystem multifunctionality of *Camellia oleifera* forests.

Many previous studies have suggested that plant (Guo et al., 2025) and microbial diversity (Saleem et al., 2019) are drivers of soil health. This study indicated that alfalfa mulching-triggered increase in understory vegetation species richness favored soil health (Spearman *r* = 0.57–0.62, *p* < 0.05, Figure 5), but the alfalfa mulching-triggered simplification of soil bacteria (Supplementary Figure S3) likely enhanced soil health (bacterial Chao 1 and Shannon, Spearman *r* = −0.62–-0.51, *p* < 0.05, Figure 5). This to some extent supported recent findings that the decreased the microbial diversity accompanied by a decrease in the abundance of common pathogen in roots improved the fruit yield of *Camellia oleifera* forests (Gu et al., 2025). Furthermore, this study, for the first time, showed that microbial diversity (individual effect, 18.40%) probably played a more important role than understory vegetation diversity (individual effect, 4.15%) in shaping soil health, and microbial life-history strategy (individual effect, 17.23%) probably played a more important role than microbial community stability, microbial cross-kingdom network stability, and phylogenetic bin-based microbial assembly (individual effects, 3.21%) in shaping soil health (Figure 6a). This study also supported that grass mulching soils were copiotrophic habitats and bare control soils were oligotrophic habitats (Chen et al., 2021). The increases in water and nutrients increased copiotrophic bacteria (Wang N. et al., 2024) and decreased oligotrophic bacteria (Yang et al., 2020; Yang et al., 2024) because of their nutritional preference (Yang et al., 2024). These suggested that soil bacterial communities shifted from the slow growth strategy to the fast growth one (Yang et al., 2024). This trade-offs in life-history strategies probably favored soil health (Spearman *r* = 0.59–0.61, *p* < 0.05 for copiotrophic bacteria; Spearman *r* = −0.67–-0.55, *p* < 0.05 for oligotrophic bacteria). Consistent with previous findings, mulching enhanced the synergies (Wang N. et al., 2024) (Figures 4a,b) and stability (Kang et al., 2025) (Figure 4e) of cross kingdom network. However, they showed low individual effect on soil health (Figure 6a). Nevertheless, the enhanced soil bacterial community stability (Ding et al., 2026) probably facilitated soil health (Spearman *r* = 0.53–0.57, *p* < 0.05).

Collectively, alfalfa mulching could be recommended for improving soil physical, chemical and microbial health, and soil microbial function (individual effect, 56.99%) could be the primary leverage point of soil health of the *Camellia oleifera* forests (Figure 6a).

### Linking ecosystem multifunctionality to microbiota and plant

A recent global meta-analysis suggested that the grass mulching significantly increased the provisioning service, regulating services, and supporting services of orchard ecosystems (Hu P. et al., 2022). Our study provided evidence for the first time that alfalfa mulching enhanced the ecosystem multifunctionality of the *Camellia oleifera* forests. The relative importance of plant and microbial diversity to ecosystem multifunctionality always been a controversial hot topic. For instance, plant diversity had a greater impact on multifunctionality than soil microbial diversity did in shrubby grassland of Guizhou plateau (Ding L. et al., 2024) and wetlands of Qinghai-Tibetan plateau (Zhang C. et al., 2024), however, in this study, the individual effect of microbial diversity (19.56%) was supposed higher than the individual effect of understory vegetation diversity (12.18%) on ecosystem multifunctionality. Recently, increasing numbers of studies suggested that community assembly, diversity, network stability driven ecosystem multifunctionality (Han et al., 2021; Chang et al., 2025). This study firstly, comparatively demonstrated the relative influence of understory vegetation diversity, soil microbial function, microbial diversity, microbial life-history strategy, microbial community stability, microbial cross-kingdom network stability, and phylogenetic bin assembly on the ecosystem multifunctionality. Soil microbial function, microbial diversity, and microbial life-history strategy were supposed to predominately drive the ecosystem multifunctionality due to the individual effects of 68.2% (Figure 6b). Since the highest individual effect (32.94%), soil microbial function was considered the potentially strongest driver of ecosystem multifunctionality of the *Camellia oleifera* forests (Figure 6b). Some studies found that soil health could be improved by elevating ecosystem multifunctionality (Li X. et al., 2023), however, other suggested enhancing soil health could improve ecosystem multifunctionality (Yang R. et al., 2023). This study showed synergism between ecosystem multifunctionality and soil health in *Camellia oleifera* forests (Supplementary Figure S4), implied that alfalfa mulching was a win-win strategy. However, the underlying causal relationship still needs to be explored. Collectively, this study provides scientific evidence for the enhancing of ecosystem multifunctionality of the *Camellia oleifera* forests on subtropical karst area, via the alfalfa mulching. This also beneficial for guiding the further renovation practices of low-yielding *Camellia oleifera* forests.

## Conclusion

Meeting soil health demands while sustaining high levels of ecosystem multifunctionality remains a key challenge in agroforestry systems. Supporting our two hypotheses, this study demonstrates that alfalfa mulching offers a practical, ecologically based solution: it enhanced soil health and ecosystem multifunctionality. Notably, soil microbial life-history strategy probably emerged as a more influential driver than community stability, cross-kingdom network stability, or phylogenetic bin assembly processes on both soil health and multifunctionality. Soil microbial function was considered the potentially primary leverage point. For low-yielding *Camellia oleifera* plantations, rather than focusing exclusively on vegetation management, renovation efforts should shift from above-ground management toward both above- and below-ground communities through alfalfa mulching. Practices that improve soil microbial function should also be carefully considered.

## Data Availability

The amplicon raw data are deposited in the FigShare repository, accession number: 10.6084/m9.figshare.30187921 and 10.6084/m9.figshare.30187990. Metagenomic raw data are deposited in NCBI with the project name of PRJNA1433700.

## Footnotes

1
https://github.com/dlltargeting/MicrobUIQ
