# Supplementary figures and images for: Alfalfa mulching improved soil health and ecosystem multifunctionality of Camellia oleifera forests on a subtropical karst brae
Source: Front Microbiol. 2026 Apr 14;17:1770805. doi: 10.3389/fmicb.2026.1770805 (PMC13121140; doi:10.3389/fmicb.2026.1770805)

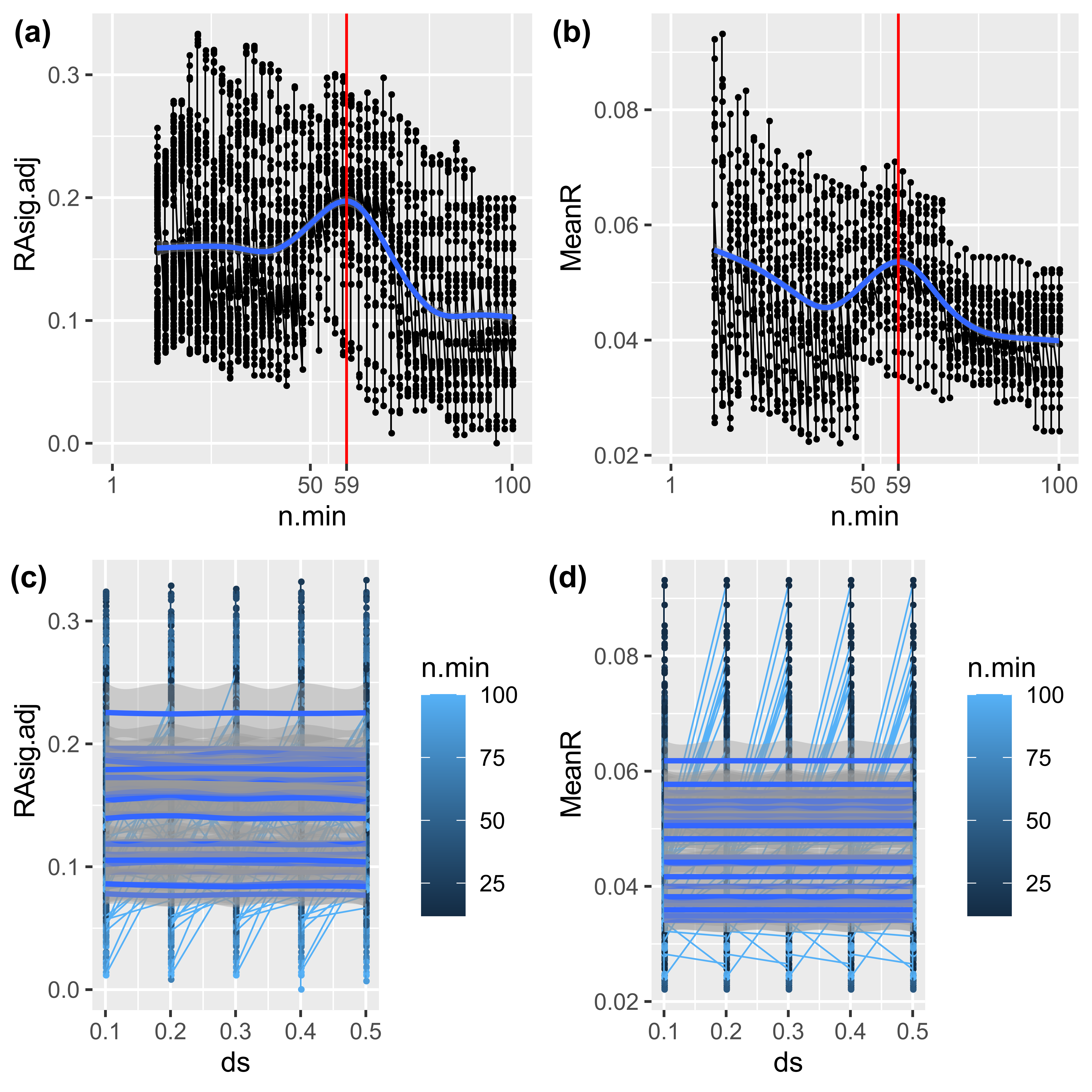

Supplement: Supplementary file 1 [file Image_1.tif]

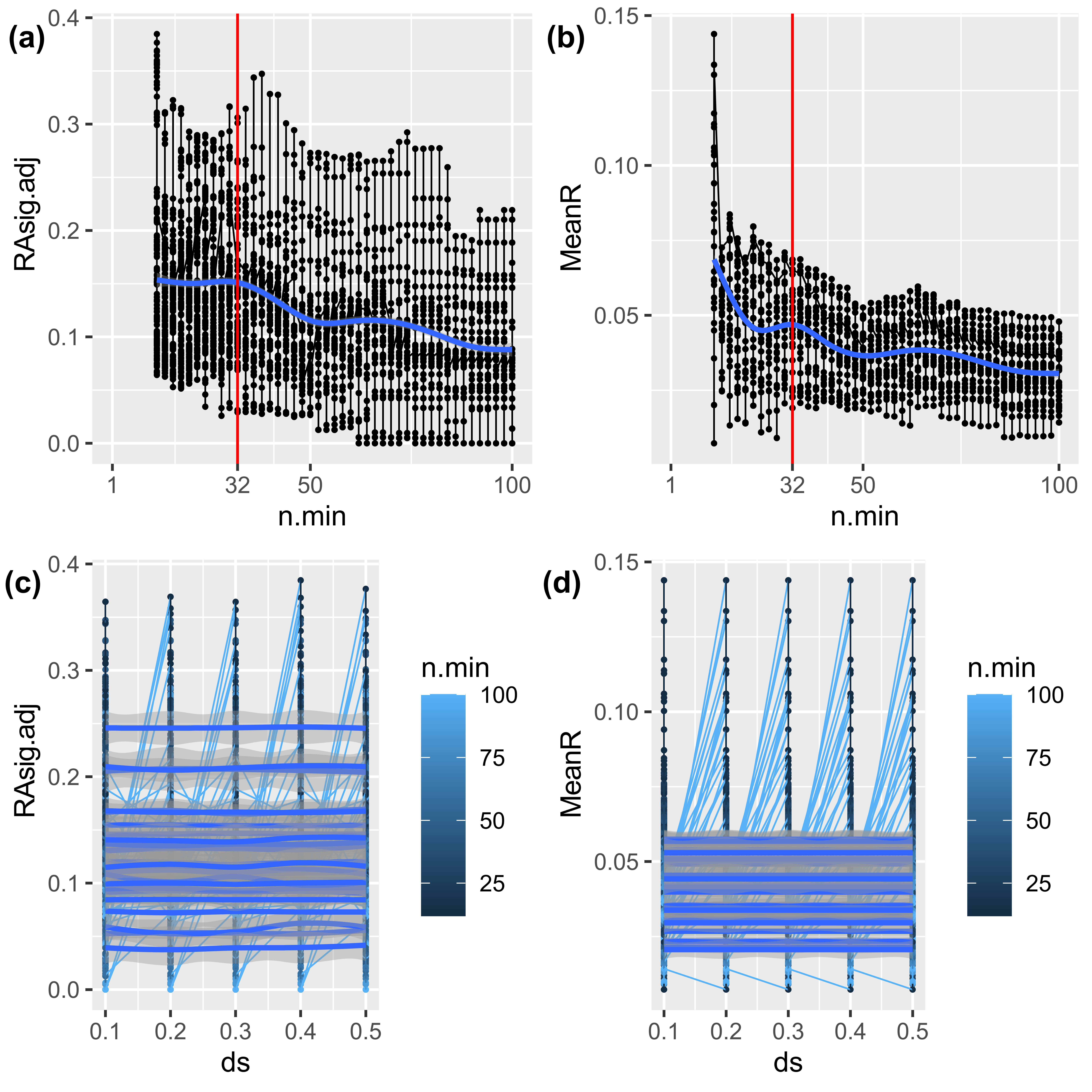

Supplement: Supplementary file 2 [file Image_2.tif]

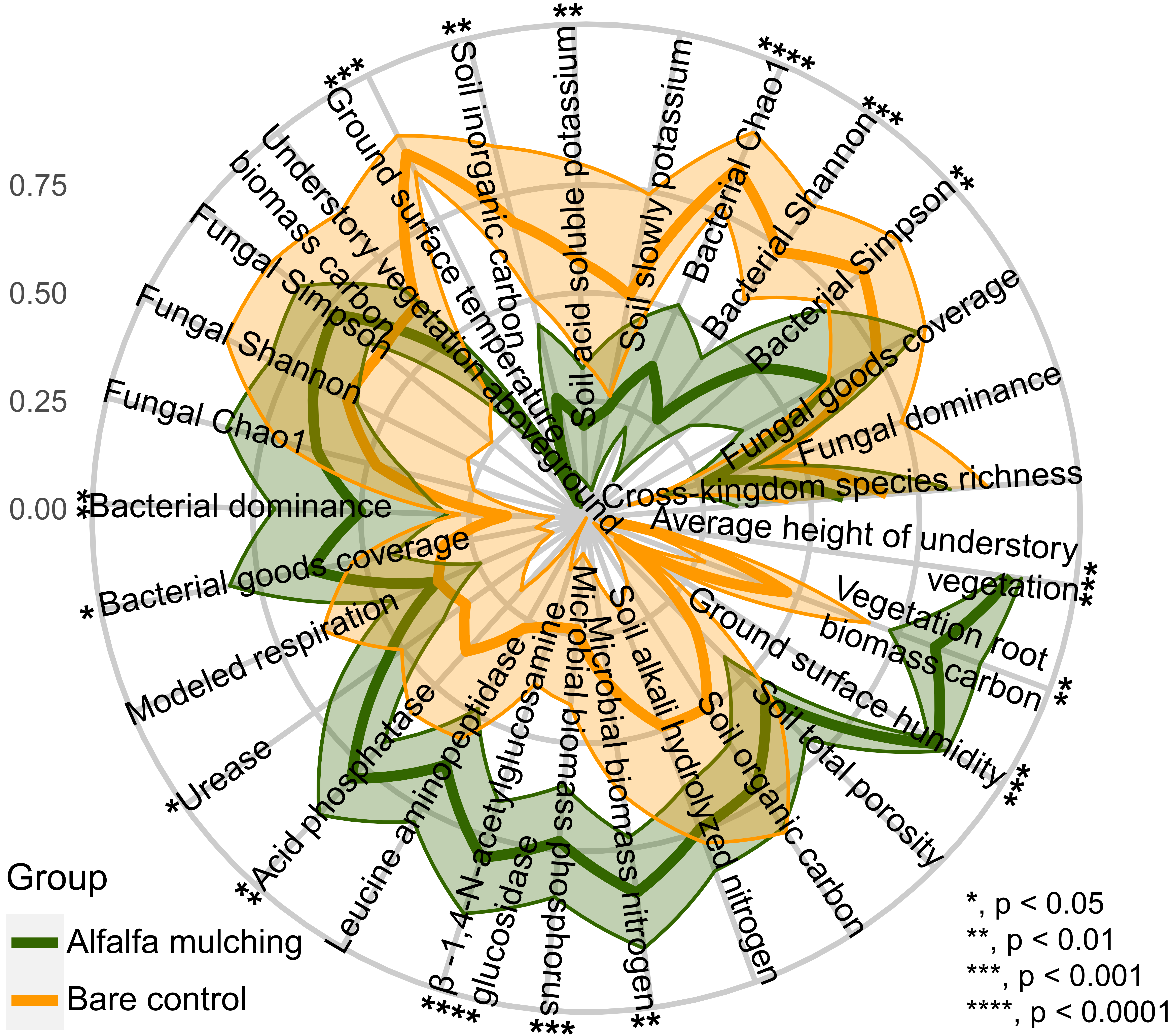

Supplement: Supplementary file 3 [file Image_3.tif]
